# Establishing the root causes of unsafe behaviors among construction workers: an integrative interpretive structural modeling analysis

**DOI:** 10.1038/s41598-023-31793-4

**Published:** 2023-04-28

**Authors:** S. Sathvik, L. Krishnaraj, Bankole Ostia Awuzie

**Affiliations:** 1 Department of Civil Engineering, SRM Institute of Science & Technology, Kattankulathur, 603203 India; 2 School of Construction Economics and Management, University of Witwatersrand, Jan Smuts Avenue, Braamfontein, Johannesburg, 2050 South Africa

**Keywords:** Civil engineering, Human behaviour

## Abstract

There has been a significant decline in worker productivity at construction sites globally owing to the increase in accidents and fatalities due to unsafe behavior among workers. Although many studies have explored the incidence of unsafe behaviors among construction workers, limited studies have attempted to evaluate the causal factors and to determine the root causes. An integrative interpretive structural modeling analysis of the interrelationships that exist between these causal factors established from relevant literature was conducted in this study to determine the root factors hence bridging this gap. Fifteen causal factors were identified through literature review, and the nature of interrelationships between them was determined using interpretive structural modeling (ISM) and a Cross-impact matrix multiplication applied to classification (MICMAC) analysis. Data was obtained from a purposively selected cohort of experts using semi-structured interviews. The emergent data was subsequently analyzed using the ISM and MICMAC analysis to ascertain the interrelationships between the causal factors. The results of the study showed that age, sleep quality, degree of interaction and workers’ skillsets were the root causes of unsafe behavior among construction workers. Besides engendering the establishment of the root causes of unsafe behavior among construction workers, the results of this study will facilitate the prioritization of appropriate solutions for tackling the menace.

## Introduction

The construction industry involves high degrees of risk, and the safety of workers on construction sites remains crucial to successful project delivery. This has raised the need for prioritizing and implementing effective health and safety procedures for workers within construction organizations and projects. However, despite the development and implementation of various safety regulations and guidelines, accidents and fatalities still occur within project delivery environments^1–3^. One major contributing factor to such incidents is unsafe behavior among construction workers^4,5^.

Numerous studies have attempted to explain the safety behavior of construction workers based on cognitive perspectives^6^. Also, recent research indicated that some individual background characteristics, such as individual and physiological factors, affect the safety behavior of workers. For example, safety knowledge and motivation are referred to as person-related factors that are strongly influence safety performance behaviors^7,8^.

Unsafe worker behavior has been described as actions or decisions made by workers, which increases the risk of injury or harm to themselves or others^9^. Such behavior has been identified as a major contributor to the increasing levels of non-compliance with the extant health and safety regulations and poor safety climate performance, especially in developing countries like India where labor intensive construction practices persist^10^. Acknowledging the significant contribution of unsafe construction worker behavior to the prevalence of unsafe working practices on construction sites, different scholars have sought to identify the causes of such behavior among workers^11^. Factors like age, gender, body mass index, alcohol consumption and smoking, level of education, training and experience, health status, psychological behavior, worker location, degree of interaction, worker’s skill type, work environment, sleeping habits, sleep onset, sleep quality, have been identified by scholars as contributing to unsafe behavior among construction workers^12^. However, the impact of the identification of these causal factors on the management or mitigation of the incidence of such behavior remains debatable in the face of the increasing degree of non-compliance witnessed therein. Some of these factors have been known to serve as triggers for other causal factors. Therefore, curbing such factors would obviously culminate in the mitigation of other factors. An understanding of the nature of interrelationships existing between these previously mentioned causal factors would assist in determining the root factors and facilitate the development and prioritization of relevant solutions.

Therefore, such determination of the root causes of unsafe behavior among construction workers remains crucial for developing effective accident prevention strategies on construction sites, and indirectly resulting in improved construction labor productivity within the sector. Suffice to state that by addressing these underlying factors, employers can create a safer work environment and ensure the well-being of their workers^13^. This study seeks to contribute to the extant body of knowledge by establishing the relationships existing between these factors and determining the root causes of unsafe behavior among construction workers in developing country contexts using India as an exemplar. To achieve this objective, this study sets out to elicit responses to the following research questions.

- RQ1. What is the nature of interrelationship existing between causal factors of unsafe behavior among construction workers in India?
- RQ2. What root causal factors were observed from the interrelationships developed in RQ1?
- RQ3.What insights can be gained from analyzing and visualizing these factors using integrative interpretive structural modeling (ISM) to improve worker productivity by reducing accidents and fatalities?

The objectives of this study are as follows:

- O1. To establish the nature of interrelationships existing between factors causing unsafe behavior among construction workers using an ISM and MICMAC analysis.
- O2. To determine the root causal factors of unsafe behavior among construction workers.
- O3. To analyze and visualize how cognitive factors influence worker productivity in a complex organizational setting using integrated interpretive structural modeling (ISM).

The remainder of the paper is organized as follows. “Literature review” consists of a review of literature relating to the various causal factors of unsafe worker behavior. “Methodology” shows the methodology used for data collection and analysis. "Results" comprises the results from the study. "Discussion" discusses the results of the study while considering the results from similar studies. "Implication" outlines the implication emerging from the study's results. "Conclusions" outlines the conclusion from the study.

## Literature review

Achieving zero fatalities and accidents in the construction industry is hampered by worker behavior. Scholars have highlighted the increasingly significance of a positive safety climate on improved construction productivity^10^. Similarly, studies have examined the nexus between worker productivity, the construction-worker safety, and construction project performance^13,14^. This rising interest is indicative of the increasing importance of this field in the contemporary construction industry^15^.

The literature focusing on the reduction of unsafe human behavior among construction workers is replete with descriptions of its primary causes^5–7,10,11,15–17^. These primary factors as elicited from literature are discussed in the subsequent section.

### Factors causing unsafe construction worker behavior

#### Age

Age was found to be the most important factor that affects the productivity of the construction workers. Age may be associated with a decline in physical and cognitive abilities, but expertise and experience can make up for this. Furthermore, employers should provide their employees with workplace flexibility and support to ensure they continue to contribute to the workforce and maintain their productivity over the years. In addition, employers must understand the value of their aging employees and to ensure they receive the resources and support they need to succeed. Age-dependent cognitive and behavioral differences exist among construction workers. According to the bureau of labor statistics, workers can be classified into young (21–30 years), middle (30–45 years) and old (45–60 years). As construction workers age, they assume familial and societal responsibilities, which impairs their ability to make sound judgments^18^. Teenage employees may exhibit impulsive behavior and irrational decision-making in emergency situations owing to their youth and lack of experience. Furthermore, the effectiveness of sleep increases with age; therefore, sleep is highly dependent on age^19^. Therefore, supervisors should use conditional judgment and assess the situation before taking action with young workers^3^.

#### Gender

Owing to the evident disparities in gender, male and female workers may behave considerably differently in the event of an accident. To be highly productive at work, men are more likely than women to work to the best of their skills. However, female workers like to work swiftly and finish the task at hand. The success or failure of a construction project may vary based on the gender owing to varied construction behaviors. Therefore, gender is crucial throughout the construction process.

Furthermore, female workers have a different body behavioral cycle than male workers, and their sleep hours are even fewer when they take on domestic obligations, which may be a contributing factor to the variation in the quality of sleep between male and female workers^20^. While sleeping for longer periods of time, with shorter sleep-onset latency and higher sleep quality than males, women have lower sleep quality. In addition, females complain about sleep more frequently than males. However, both men and women have reduced slow-wave sleep as they age^21^. A worker's psychological state may change as they age, and they may get more fatigued from getting less sleep, which reduces their productivity at work. Therefore, it is necessary to adjust the work schedule of the female workforce to accommodate their needs.

#### Body mass index

Because BMI and sleep quality are related, BMI is a significant incidental factor^22^. BMI is calculated based on a person's weight and height. A construction worker's weight often falls within the normal or healthy range. A BMI between 20.1 and 25.9 indicates overweight, whereas a BMI of 26.1 or more indicates obesity. The BMI of construction workers should be within the normal range for adequate sleep^23^.

#### Alcohol consumption and smoking

The consumption of substance, such as drugs, alcohol, and tobacco, can negatively impact sleep and overall health, which can lower productivity and wellbeing at work. Alcohol, in particular, can interfere with sleep patterns and quality^24^. When consumed before bed, alcohol can make falling and staying asleep difficult, which changes the stages of the sleep cycle, leading to lighter, less restful sleep. Furthermore, prolonged or frequent substance use can have negative long-term effects on health, including increased risk of sleep disorders, cardiovascular disease, and other health problems^25^. For construction workers, who often work long hours and perform physically demanding tasks, maintaining good sleep hygiene and avoiding substances that can disrupt their sleep is important. Therefore, they need to ensure they have the energy and focus needed to work safely and effectively^23^.

#### Education level

Less educated employees are more likely to exhibit poor workplace productivity, while highly educated workers are more likely to recognize good productivity and provide better results. Additionally, insufficient sleep can impact the conduct of employees of all educational levels as well as their productivity^17^, which are significantly related. The length of various construction activities is further influenced by the education level^20^. Higher educated construction employees exhibit improved performance and productivity using intelligent work practices; however, employees with less education perform poorly and are less productive.

#### Training and experience

Providing workers with training and education on important topics such as sleep and productivity is essential for their health and safety on the job. Research has shown that worker behavior is closely linked to their training; therefore, providing quality training and education is critical for promoting positive behavior and habits on the job^20^. Furthermore, emergency preparedness training is crucial for ensuring the safety of workers^26^. Therefore, by providing workers with the knowledge and skills they need to respond to emergencies, companies can ensure that they are able to work safely and effectively, even in challenging conditions.

In addition, it is important that workers receive adequate supervision and support at the job. Workers whose supervisors provide in-depth training and experience tend to be more productive; therefore, investing in appropriate support can positively impact the overall work quality in the construction industry^23^. Overall, providing training, education, and support to construction workers is critical for promoting their health and safety, as well as the quality and productivity of their work. Therefore, companies can create a safe, highly productive work environment for their workers^27^.

#### Health status

Health status plays an important role in determining a person's overall wellbeing and ability to perform physically demanding tasks, such as those required in the construction industry^28^. Factors such as disease and intoxication can affect a worker's physical ability, increasing the risk of accidents on the job. Physiological factors, such as age, can drastically impact a worker's ability to respond effectively during an emergency^29^.

Construction workers under the influence of alcohol or drugs are prone to making mistakes or acting impulsively, which can increase the risk of accidents^30^. Similarly, workers younger than 21 or older than 50 years are likely to be impaired than those of other age groups, resulting in increased risk for accidents^31^. Overall, companies need to consider the health status and physiological factors of their workers when designing and implementing safety measures and training programs. Therefore, they can help ensure that workers can safely and effectively perform their jobs, even in challenging conditions^18^.

#### Psychological behavior

Psychological behavior and cognitive processes are influenced by emotions and feelings, especially during times of crisis. It has been observed that when crowds are present, occupants are nonjudgmental and conform to their surroundings rather than immediately evacuating the site^23^. Therefore, performing construction tasks in a calm environment allows workers to accurately evaluate the workplace^32^. A study shows that when workers exhibit low productivity, it can be attributed to panic in a retrospective, contemporary, or anticipatory manner by and to different group roles in varying degrees^33^.

#### Worker location

One of the unintentional behavioral aspects during construction is the worker location at the job. If a worker is present at the place of employment, he/she should display a manageable range of over 7 h of sleep^21^. The worker would, however, uncontrollably suffer a range of fewer than 7 h of sleep, as well as a disordered stage of sleep if he/she is placed distant from the site of employment. Workers behave differently and give off varied sleep-range signs depending on where they are^34^. At a location close to the site, employees personally verify the information on the work schedule. Workers in remote locations are not aware that work is being done until they receive the productivity loss and decline in the quality of the output^2^.

#### Degree of interaction

Effective interaction, which is feasible between employees in any language, disseminates the knowledge required for staff to perform their duties and nurtures bonds based on commitment and trust^28^. The potential for productivity and smooth operation of an organization is determined by the effectiveness of its interactions. The importance of interaction in educating construction workers about the value and impacts of sleep cannot be overstated. Therefore, interactions can increase the productivity of workers who have healthy lifestyles and get enough sleep^35^. The impact of sleep on work life and employee behavior must be discussed between managers and staff members to achieve the necessary productivity.

#### Worker’s skill type

Based on their skillsets, construction workers can be classified as unskilled, semiskilled, and skilled workers.

##### Unskilled workers

Unskilled workers lack specific abilities. They are highly unlikely to have advanced degrees and or any training on the value of sleep. These workers typically carry out straightforward jobs that need physical effort and exertion without requiring judgement^36^. As technology advances, the number of jobs available for unskilled workers decreases.

##### Semiskilled worker

Although semiskilled workers do not need advanced training, the activities they perform require greater expertise than those of unskilled individuals. Semi-skilled construction workers often hold a high-school diploma or its equivalent, and they have some understanding of how sleep affects their lives^29^. Although they do not need advanced abilities, they do need to be able to keep track of and perform repeated activities. These skills are more easily transferable to different fields than those of unskilled workers.

##### Skilled worker

A skilled worker is equipped with knowledge or abilities. Workers in these professions are skilled in their trades and can use judgement. Unlike unskilled and semi-skilled workers, most professionals having college degrees and understand the importance of sleep. The need for trained labor increases as specialized talents become more crucial. Construction workers with more specific training, such as doctors, would fall under a different group of professionals.

#### Work environment

Factors such as temperature, noise, and light in a sleep environment can affect the quantity and quality of sleep^32,37^. A person should put away any distractions or stress-inducing elements in the sleeping environment to get the best possible sleep. Researchers have shown that humans’ sleeping temperature varies significantly. Therefore, the ideal temperature for a bedroom cannot be established. When the temperature is the most pleasant for the individual, sound sleep can be attained^38^. Extreme temperatures, however, frequently cause sleep disturbances in sleeping situations. During the sleeping environment, temperature changes are more frequent. However, REM may become impossible in severe chilly conditions, disrupting sleep.

#### Sleeping habits

While bad sleep habits frequently cause changes to sleep quality, good sleep can improve it. While the sleep quality of a few employees is good, others do not get sufficient rest^39^. Construction workers are more prone to developing restless leg syndrome, which causes tingling, discomfort, and uncontrollable leg movements while sleeping^38^. Worker performance, mood, and health are all improved by good sleep habits. Therefore, numerous diseases and disorders are likely to develop in those who do not get sufficient, regular, high-quality sleep.

#### Sleep onset

Construction workers are increasingly less productive owing to poor sleep hygiene caused by lifestyle choices or medical conditions^14^. Sleep apnea, insomnia, or narcolepsy are some sleep disorders that affect construction workers, which can induce neuro-psychophysical consequences such as sadness, anxiety, heart problems, hallucinations, and mood swings^40^ and affect the well-being and productivity of construction employees on the job site. Insomnia is a common sleep disorder that significantly affects a person's physical and mental health, including their ability to safely perform job-related tasks^18^. In the context of construction work, insomnia can be particularly concerning as it can increase the risk of accidents and injuries on the job. Several studies have explored the relationship between insomnia and construction safety, examining factors such as fatigue, impaired cognitive function, and reduced reaction time^41^. Christian and Ellis^34^ described two types of insomnia with varying effects on human capability: total and partial insomnia. Diverse authors agree on the debilitating effects of insomnia on a person's motor, cognitive, and emotional abilities, thereby impairing their ability to engage in daily activities^1,8,34^. Killgore^8^ argued that although insomnia inhibits creativity and cognition, it does not affect rule-based reasoning, decision-making, or task planning. Similarly, Christian and Ellis^34^ explained the instances of workplace deviance owing to workers losing self-control and becoming more hostile owing to insufficient sleep. Eksander et al.^1^ acknowledged that lack of sleep contributes to the social, psychological, and cognitive factors that negatively affect construction safety among construction workers. These studies have highlighted the importance of addressing sleep problems in the construction industry to ensure a safe and healthy work environment^42^. However, some points have not been thoroughly investigated in these studies^30,31,43,44^. For example, while many studies have examined the effects of sleep deprivation on construction workers, only a few studies have specifically studied the impact of insomnia. In addition, some cultural and social factors may contribute to sleep problems among construction workers that have not been fully explored^21^.

#### Sleep quality

Several studies have demonstrated that the quality of sleep significantly influences the frequency of unsafe behavior^11^. Several factors contribute to sleep quality, including how much sleep is obtained and how much time is spent sleeping^42^. Sleep quality is generally above 90% among young healthy workers. Sleep quality of workers be at least 80% is considered normal/healthy. The inability to achieve the recommended amount of sleep (typically less than seven hours) has been referred to as poor sleep quality^8,10,34,45^. Getting quality sleep can ensure necessary physical, mental, and emotional benefits. Considering the effects, sleep may be considered a major contributor to unsafe behavior among construction workers as the productivity and performance of construction workers remains highly dependent on their sleep quality.

Following from a comprehensive discussion of the factors causing unsafe behavior among construction workers, a compilation of these factors and the sources is provided in Table 1. However, as admitted previously, a lack of studies seeking to explore the nature of the relationships between these factors persists. The lack of such studies has made it difficult to establish the root factors and to prioritise solution development. This study aims to fill this gap by examining the relationships between these causal factors using a combination of interpretive structural modelling and MICMAC analysis respectively.

**Table 1:** Factors affecting worker productivity in the construction industry.

Sl. no.	Factors	Tag	References
1	Age	A1	^3,18,19^
2	Gender	A2	^4,37,46^
3	Body mass index	A3	^3,30,43^
4	Alcohol consumption and smoking	A4	^31,44^
5	Educational level	A5	^17,20^
6	Training and experience	A6	^3,21,23,24^
7	Health status	A7	^13,42,47,48^
8	Psychological behavior	A8	^32,33^
9	Worker location	A9	^26,46^
10	Degree of Interaction	A10	^28,35^
11	Worker’s skill type	A11	^29,36^
12	Work environment	A12	^37,38^
13	Sleeping habits	A13	^38,39^
14	Sleep onset	A14	^32,40^
15	Sleep quality	A15	^25,42^

Source: Authors’ Compilation, 2023.

## Methodology

The ISM methodology is useful for dealing with complex problems and provides a clear structure of the relationships among different components in a complex system. By utilizing the knowledge and expertise of experts, the ISM method can help analyze the issues and develop a multi-level structural model that can be used to make informed decisions. The ISM model is particularly useful in prioritizing variables and effectively allocating resources. The driver-dependency clustered map created by ISM provides a clear understanding of the complex situation and hierarchical interactions among the components, which can help identify novel approaches to address the problem.

In the context of preventing unsafe acts by construction workers, ISM can be used to understudy and present the nature of interrelationships between factors that contribute to unsafe behaviors among workers, hence engendering the development of appropriate and effective interventions to curb such behavior. An ISM model can provide insights into the relationships between these factors and help understand how they interact with each other, allowing for a comprehensive approach to address the issue. Therefore, an integrative interpretive structural modeling analysis offers an invaluable tool for this study considering the objectives that it seeks to achieve.

### Data collection and analysis

Several construction-industry experts were interviewed using semi-structured interviews to achieve the research objectives. Before the interviews were conducted, an extensive literature review identified 15 causal factors that affect the productivity of construction workers. We initially contacted 65 experts, and eight of them expressed interest in participating in the study. A one hour minute question-and-answer session was scheduled with each expert. These interviews lasted for an average of 45 min and were conducted in the locations chosen by the interviewees. Table 2 summarizes the demographics of these specialists.

**Table 2:** Expert background information.

Expert	Education	Occupation	Work experience in (years)
1	PhD	Researcher	24
2	Master’s	Project manager	18
3	Bachelor’s	Contractor	15
4	PhD	Researcher	12
5	Master’s	Contractor	17
6	PhD	Researcher	20
7	Master’s	Engineer	13
8	Bachelor’s	Supervisor	19

Source: Authors’ compilation.

**Figure 1:**
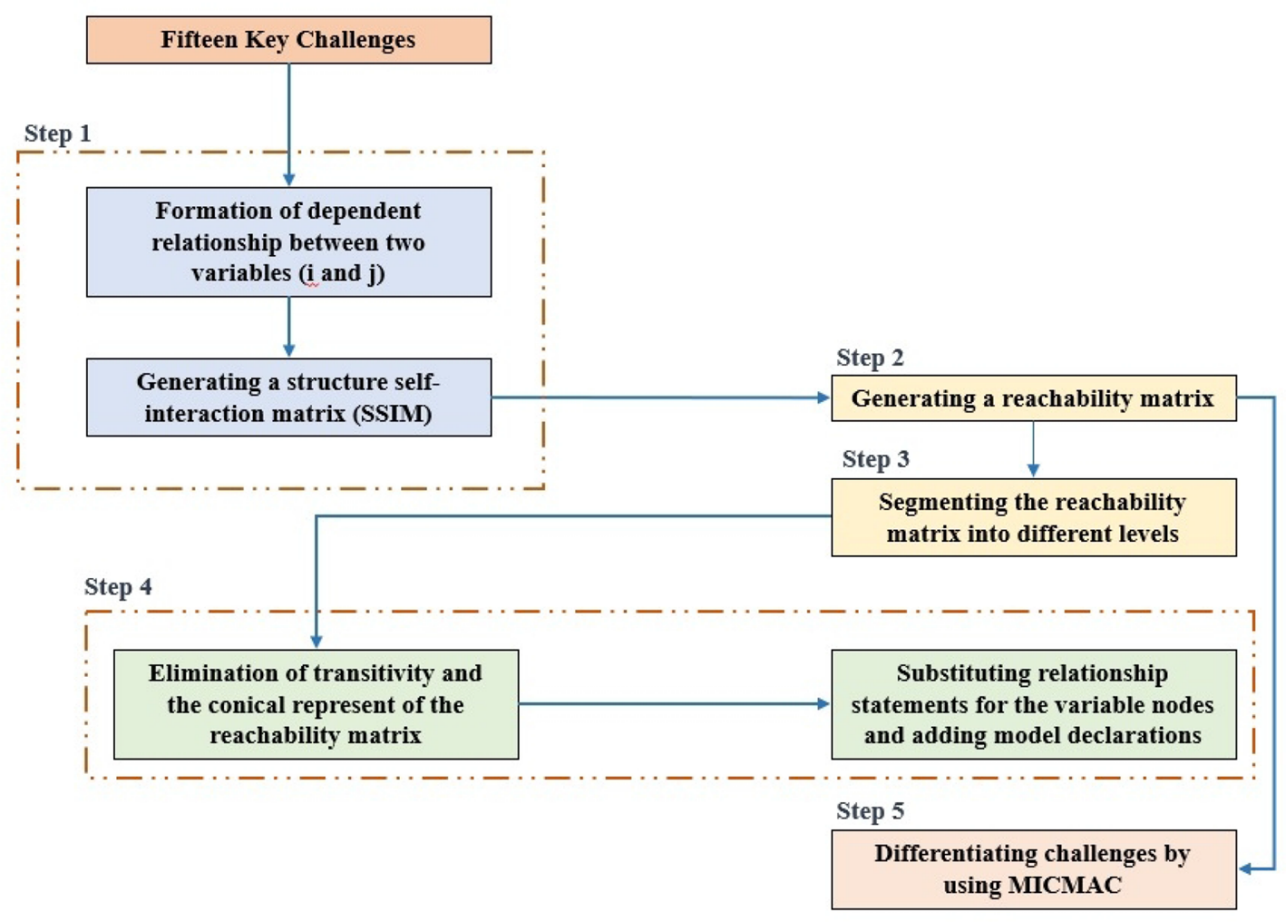
Methodology and process of ISM diagram.

The contextual relationship between causal factors was discussed during the interview sessions. Despite the complexity and non-binding nature of the interview protocol, the results were positive. To analyze the interrelationships among these causal factors, the collected answers were coded as 0 and 1 based on their interpretation of Yes or No. An ISM diagram was then created using the expert responses^11^. The emergent hierarchical structure was analyzed for transitivity using power iteration while creating reachability and adjacency matrices. According to the MICMAC analysis, each factor was classified by the power with which it drove and was dependent on another factor which is listed in Fig. 1. Consequently, ISM is a suitable method for describing existing interrelationships among factors.

#### Ethical approval

This research was approved by the Ethics Committee of the SRM Hospital and Medical Research Centre (2186/IEC/2020) and was conducted according to the principles of the Institutional Ethical Committee. Informed consent was obtained from all the participants included in this study.

### ISM methodology

In the methodology section of the ISM, nine steps are described, which can be seen in Fig. 1 and explained below.

*Step 1* A multidisciplinary expert team was formed and tasked with applying their expertise to various stages of the study, including the development of an initial reachability matrix, establishment of transitivity links, and validation of the developed structural model.

*Step 2* This literature review identified and defined the factors that hinder the influence of worker productivity. ISM comprises these factors.

*Step 3* An interpretive logic knowledge base was established through pairwise comparisons, which comprised yes/no answers for relationships among the elements along with the rationale behind the yes answers. To determine a pairwise contextual relationship between the barriers, working with the resource team was necessary.

*Step 4* An interpretive logic knowledge base was used to conduct binary pairwise comparisons. A 'yes' was defined in the interpretive logic knowledge base as '1', whereas 'no' was defined as '0'. A binary interpretation of '1' indicates that there is a contextual relationship, whereas '0' indicates that there is no contextual relationship. Pairwise comparison matrices were developed using logical interpretations for an m × m matrix, where m represents the number of barriers. This step involved the creation of a pairwise comparison matrix, which represents the initial reachability matrix.

*Step 5* Contextual relationships indicate the existence of direct relationships in the initial reachability matrix. However, a transitivity check can reveal several indirect relationships. The rule for a transitivity check is: if ‘A’ influences ‘B’ and ‘B’ influences ‘C’, then ‘A’ influences ‘C’. The interpretive knowledge base represents each identified transitive link as a 'transitive link'. Moreover, we identified the elements that provided the links. Experts contributed to the identification of transitive links with important interpretations for further analysis. Efficacious links and direct relationships are included in the reachability matrix.

*Step 6* Level-partitioning was used to determine the factor levels based on the final reachability matrix. Reachability refers to the driving factors, whereas antecedent refers to dependencies. Prior to determining the intersection set, the reachability and antecedent sets must be determined. This factor was designated as the highest level in the first iteration considering it had the same reachability and intersection sets (Level I). This iteration resulted in all factors of Levels I and II being removed from all sets and being identified, respectively. Each factor was assessed in the same manner until all factors were rated.

*Step 7* The factors identified in the previous step were incorporated into the digraphs, and the links were represented based on the final reachability matrix.

*Step 8* An interpretation was indicated in the interpretive matrix for all entries with a value of '1' in the final reachability matrix.

*Step 9* Finally, we developed an ISM model. In Step 7, the interpretation developed in Step 4 was incorporated into the digraph, which was used to develop the ISM model.

## Results

### Establishing a structured self-interaction matrix (SSIM)

Literature reviews and expert opinions have indicated that unsafe behavior among construction workers is influenced by several factors. This study aimed to compare these assessments to improve our understanding on these factors. I and J were analyzed using the structured self-interaction matrix (SSIM):

**P:** Development function i causes (determines) development function j;

**Q:** Development function i is caused (determined) by the development function j:

**R:** Development functions i and j mutually cause (determine) each other.

**S:** Development functions i and j are unrelated (independent).

When developing the SSIM, we considered the factors that affect worker productivity (Table 1). In the following examples, coding is demonstrated according to the definitions above. Codes (1, 11) and (4, 10) were assigned codes Q and P, respectively.

Although Factor A2 differs significantly from Factor A3, they are supposedly influenced by each other; therefore, code X is placed in cells (2, 3). In this cell, code O was placed because there was no correlation between A10 and code O. These factors were considered when filling the cells in all cases.

### Establishing a matrix of reachability

In SSIM, the binary numbers 1 and 0 were substituted with codes P, Q, R, and S according to the rules presented in Table 3. A reachability matrix was generated from the analysis. A reachability matrix was created based on the following rules.

- In the cases where there is an ISM field P present, insert 1 for (i,j) and 0 for (j,i).
- Substitute 0 for (i,j) and 1 for (j,i) if the ISM contains Q.
- When R is present in the ISM, insert 1 at positions (i,j) and (j,i).
- Substitute 0 in (i,j) and (j,i) where S exists.

**Table 3:** A matrix of factors with structured self-interactions.

S. no.	Factors	15	14	13	13	11	10	9	8	7	6	5	4	3	2
1	Age	S	P	Q	S	P	Q	P	S	P	Q	P	Q	Q	R
2	Gender	R	Q	S	P	X	Q	P	S	P	S	P	Q	P	
3	Body mass index	R	P	S	S	Q	S	P	S	P	P	S	Q		
4	Consumption of alcohol and smoking	R	Q	P	P	S	S	P	Q	S	P	P			
5	Educational level	R	P	Q	P	Q	S	R	Q	Q	Q				
6	Training and experience	P	R	Q	R	P	Q	Q	P	S					
7	Status of health	R	P	Q	R	S	Q	P	Q						
8	Psychological behavior	R	P	P	R	Q	Q	S							
9	Worker location	P	P	Q	R	S	S								
10	Interaction	R	Q	P	P	S									
11	Workers type	P	R	P	Q										
12	Environment at work	S	R	P											
13	Sleeping habits	R	Q												
14	Movement of sleep onset	S													
15	Sleep quality														

P: Development function i causes (determines) development function j;

Q: Development function i is caused (determined) by the development function j:

R: Development functions i and j mutually cause (determine) each other;

S: Development functions i and j are unrelated (independent).

As transiting networks moved between nodes, tight-knit communities were observed (clusters, subgroups, and cliques). Ratios can be calculated by dividing the number of closed triplets observed by the number of open triplets that are possible in a graph to determine the ratio. A transitivity-theory concept was incorporated into the final reachability matrix. In addition, the system was examined in terms of its driving force and dependence.

### Final reachability levels

The factors were partitioned into levels based on the analysis of the factors to identify a hierarchy of factors. Final reachability matrices contain both antecedent and reachability factors of 1 as an antecedent. Furthermore, an antecedent set was created when a factor's column contained a value of 1are presented in Table 4. All factors were intersected in this step to determine their levels. In the ISM structure, reachability and intersection sets were common factors. There are five levels of classification for factors A15: 1, 2, 3, 4 and 5. Methods that cannot reach lower factors are not possible. Therefore, for subsequent iterations, these steps need to be repeated.

**Table 4:** Matrix of final reachability.

Factors	1	2	3	4	5	6	7	8	9	10	11	12	13	14	15	Driving power
A1	1	1	1	1	0	0	0	0	0	0	0	0	0	1	1	6
A2	1	1	1	1	0	0	0	0	0	0	1	1	0	1	1	8
A3	1	1	1	0	0	0	0	0	1	1	0	1	1	1	1	9
A4	1	1	1	0	1	0	0	0	0	0	0	0	0	1	1	6
A5	1	1	1	0	1	0	0	1	0	0	0	0	0	1	1	7
A6	1	1	0	1	1	1	1	1	0	0	0	0	1	1	1	10
A7	1	1	0	0	1	1	1	0	1	0	0	1	0	1	1	9
A8	0	0	1	0	1	1	1	1	0	1	0	0	1	1	1	9
A9	1	1	1	1	1	1	1	0	1	0	0	0	0	1	1	10
A10	1	1	1	1	1	1	1	0	1	1	1	0	0	1	1	12
A11	1	1	0	1	1	1	1	1	0	1	1	0	0	1	1	11
A12	1	1	1	1	1	1	1	0	1	1	0	0	0	1	1	11
A13	1	1	0	1	1	1	1	1	0	0	1	1	1	0	0	10
A14	1	1	1	0	0	0	0	0	1	1	0	0	0	1	1	7
A15	1	1	1	0	0	0	0	1	0	0	0	0	0	1	1	6
Dependence power	14	14	11	8	10	8	8	6	6	6	4	4	4	14	14	

### Establishing hierarchical divisions

A hierarchical structure can be used to partition factors based on their level and reference guidelines to identify the underlying cause of worker productivity among Indian construction workers.

A reachability matrix was used to assign antecedent and reachability to each factor. In R(Si), Si is included along with the set of factors it influences. The antecedent set includes *Si* and the factors that address *Si,* denoted as *A*(*S*_*i*_). Two sets intersect when R(Si) = R(Si)A(Si), where the intersection represents the interaction between the two sets. The ISM hierarchy places the variables with the fewest elements in the reachability and intersection sets at the top positions. A factor that leads to another factor is not included in the list of top-level factors. Once the factors are identified, they are removed, and the process is repeated for the next level. Table 5 presents a hierarchical structure that illustrates the interrelationships among factors that influence worker productivity according to ISM diagraph listed in Fig. 3.

**Table 5:** Worker productivity and its antecedents.

Level	Reachability set	Antecedent set	Intersection set
1	A1, A2, A14, A15	A1, A2, A3, A4, A5, A6, A7, A8, A9, A10, A11, A12, A13, A14, A15	A1, A2, A14, A15
1	A1, A2, A14, A15	A1, A2, A3, A4, A5, A6, A7, A8, A9, A10, A11, A12, A13, A14, A15	A1, A2, A14, A15
1	A1, A2, A14, A15	A1, A2, A3, A4, A5, A6, A7, A8, A9, A10, A11, A12, A13, A14, A15	A1, A2, A14, A15
1	A1, A2, A14, A15	A1, A2, A3, A4, A5, A6, A7, A8, A9, A10, A11, A12, A13, A14, A15	A1, A2, A14, A15
2	A3, A7	A3, A4, A5, A6, A7, A8, A9, A10, A11, A12, A13	A3, A7
2	A3, A7	A3, A4, A5, A6, A7, A8, A9, A10, A11, A12, A13	A3, A7
3	A4, A5	A4, A5, A6, A8, A9, A10, A11, A12, A13	A4, A5
3	A4, A5	A4, A5, A6, A8, A9, A10, A11, A12, A13	A4, A5
4	A6, A8, A9, A10	A6, A8, A9, A10, A11, A12, A13	A6, A8, A9, A10
4	A6, A8, A9, A10	A6, A8, A9, A10, A11, A12, A13	A6, A8, A9, A10
4	A6, A8, A9, A10	A6, A8, A9, A10, A11, A12, A13	A6, A8, A9, A10
4	A6, A8, A9, A10	A6, A8, A9, A10, A11, A12, A13	A6, A8, A9, A10
5	A11, A12, A13	A11, A12, A13	A11, A12, A13
5	A11, A12, A13	A11, A12, A13	A11, A12, A13
5	11, 12, 13	11, 12, 13	A11, A12, A13

### MICMAC analysis

As shown in Table 6, a final reachability matrix was used to calculate the driving and dependence powers. Figure 1 presents an analysis of these powers, where x and y represent the driving and dependence powers, respectively. In particular, the diagram contains four quadrants: linkage, driver, dependent, and autonomous.

**Table 6:** Dependence and driving powers of factors.

Level	Factor	Driving	Dependence
1	Age	6	14
1	Gender	8	14
1	Sleep onset	7	14
1	Sleep quality	6	14
2	Body mass index	9	11
2	Alcohol consumption and smoking	6	8
2	Training and experience	10	8
2	Health status	9	8
3	Educational level	7	10
4	Psychological behavior	9	6
4	Worker location	10	6
4	Degree of Interaction	12	6
5	Worker’s skill type	11	4
5	Work environment	11	4
5	Sleeping habits	10	4

**Figure 2:**
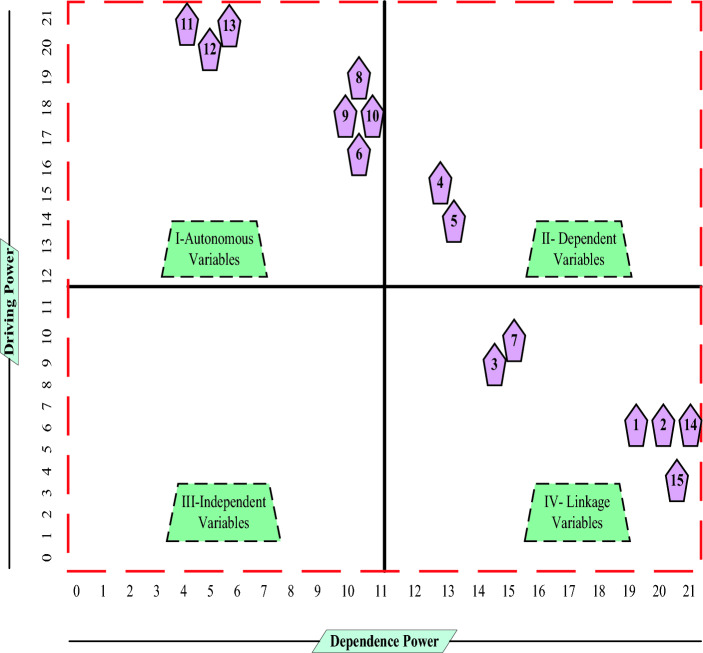
Factors of driving and dependence powers.

Several autonomous factors do not have strong drivers and are low dependents, and hence, are disconnected from the system. They differ from other system factors in a way that they cannot influence other system factors. The familiarity of a worker with the work environment as well as the level of his or her education were classified as independent, but not autonomous factors. Age, gender, sleep quality, sleeping habits, body mass index, and health status comprise the dependent cluster of factors with weak driver powers but high dependence power. A weak driving power exists in the dependent factors, whereas a strong dependency power exists in the dependent factors. Therefore, addressing and resolving other factors is necessary before making any interventions. Total two factors are classified in Fig. 2 as belonging to the linkage cluster. An influencing factor exhibits a high driving and dependence powers. All actions related to these factors also affect the remaining factors, resulting in a cascade of feedback effects. A strong correlation was observed between the psychological behavior, worker location, degree of interaction, worker’s skill type, work environment, and sleep quality as independent variables with weak dependence power. In addition, the familiarity of the workplace environment, employee location, and employee education level are also grouped into autonomous and independent clusters, as shown in Fig. 2. However, these clusters are considered autonomous since they are governed by relatively strong forces. The factors exert influence over other factors and play a significant role in system operation.

### Interpretive structural modeling

**Figure 3:**
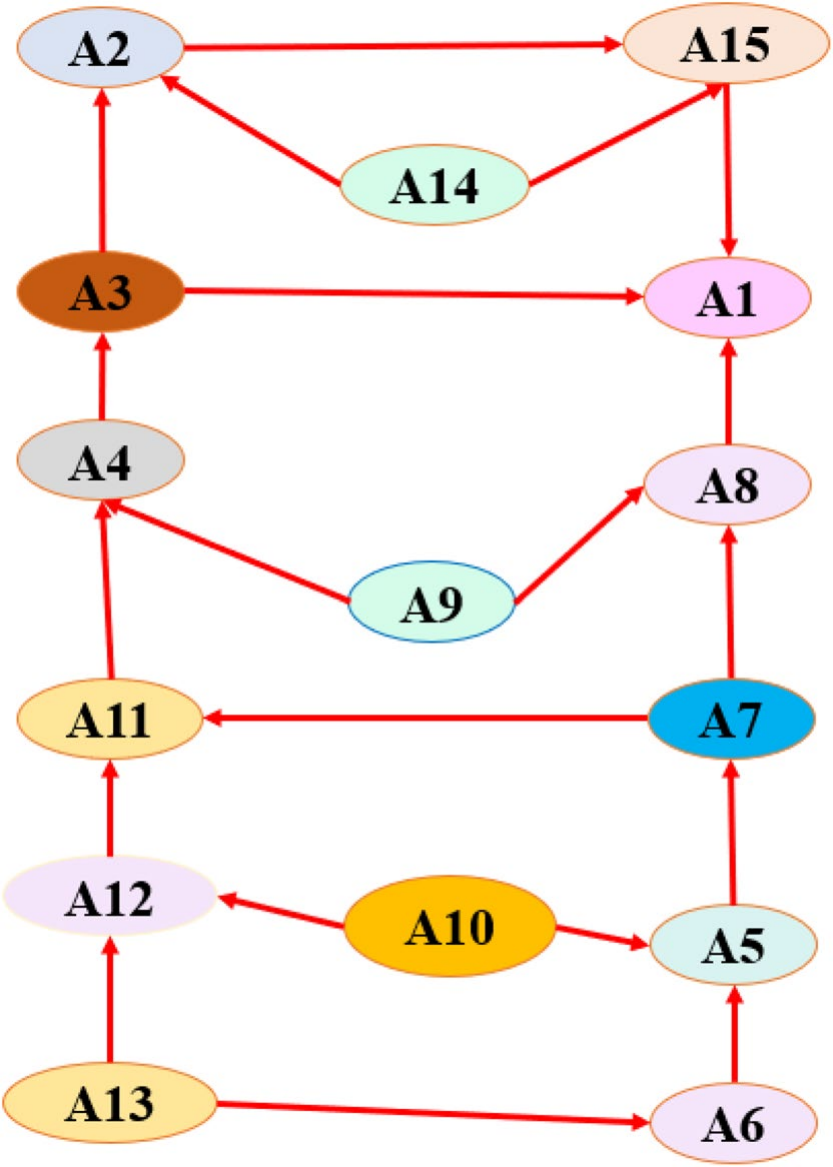
Interrelationship between the fifteen challenges.

The final reachability matrix was used to determine the reachability, antecedents, and intersections of each barrier. Factors that influence worker productivity can be categorized into the following five levels using the ISM hierarchy: Level 1 = {A1, A2, A14, A15}, Level 2 = {A3, A7}, Level 3 = {A4, A5}, Level 4 = {A6, A8, A9, A10}, and Level 5 = {A11, A12, A13 shown in Fig. 3. Worker productivity is directly impacted by the following four factors: (A1): age, gender, sleep onset, and sleep quality. The second level (indirect factors) includes a variety of genetic factors. Worker’s productivity is directly impacted by these factors. There are four indirect factors in levels 2 and 3: BMI (body mass index), health status, alcohol consumption and smoking, and education level. These indirect and direct factors are interconnected. These seven root factors are identified at levels 4 and 5: A6 (trained and experienced employees), A8 (physiological behavior), A9 (location of employees), A10 (degree of interactions), A11 (worker’s skill type), A12 (work environment), and A13 (sleep quality). Only three factors can significantly influence the incidence of unsafe behavior among construction workers, namely A11, A12, and A13, hence serving as root causal factors.

## Discussion

According to our study, unsafe worker behavior, which hinders productivity levels, are caused by age, gender, body mass index, alcohol consumption and smoking, educational level, training and experience, health status, psychological behavior, worker location, degree of interaction, worker’s skill type, work environment, sleep quality and sleep onset symptoms on safety compliance, safety participation, and minor injuries and mediating effects of workplace cognitive failure as well as for productivity of workers and Fig. 3 is showing the interrelationship between workers productivity. Among all these factors, sleep duration and age factors mainly affect worker productivity. According to our study, construction workers with insomnia are more likely to experience cognitive failures owing to a decrease in compliance with safety protocols and procedures, decline in voluntary participation in safety promotion within the organization, and higher likelihood of minor injuries^45^. Additionally, construction workers who reported not feeling well-rested upon waking were less likely to comply with safety regulations; however, this effect was not mediated by workplace cognitive impairments. No relationship was observed between sleep duration and any safety-related outcome^13,34^.

Construction workplaces should consider the quality of sleep of employees when preventing risky behavior and workplace accidents. We observed that employee sleep predicted self-regulatory behavior at work. Insomnia symptoms predicted cognitive failures, safety behaviors, and minor injuries at work, indicating that sleep affects work behavior via the cognitive components of self-regulation. Previous research has demonstrated that insomnia symptoms can affect safety-related outcomes, including workplace injuries^21^. In this work, we extended the previous work by considering the workplace cognitive failure as a self-regulation mechanism associated with safety compliance and participation^24^. Moreover, the enduring association between sleep and long-term brain changes was observed across time lags of up to 6 months with literature and past research^12^.

As a result, adequate sleep is associated with improved cognitive performance (e.g., reduced cognitive failures) at work^15^. Because measurements were collected at different points in time, our findings are less likely to be influenced by common method bias. In addition, our statistical conclusions are highly valid when based on the relationships among variables over time^14^. However, sleep insufficiency did not appear to be mediated by workplace cognitive failure and did not influence participation in safety or minor injuries^28^. Consequently, the mechanisms underlying the link between sleep insufficiency and safety compliance are currently unknown^34^. This relationship may be explained by different aspects of self-regulation. Workers who lack sleep are highly likely to defy social dilemmas and put their own interests above the interests of their co-workers or the company^49–53^.

## Implication

### Practical implications

First, the study highlights the need for organizations to assess the causal factors most critical to mitigating the incidence of unsafe behaviour among their workers. By using ISM analysis, organizations can identify the root causal factors and develop interventions that address them.

### Theoretical implications

First, the study contributes to our understanding the complex and interconnected nature of factors. Using ISM analysis, the study revealed how different causal factors of unsafe behaviour are interrelated and influence each other. This highlights the need for a more nuanced and integrated approach to understand these factors and their impact on construction worker health and safety as well as productivity.

Fourth, the study underscores the importance of taking a holistic approach to productivity improvement, which means considering not only factors that are most critical, but also how other factors, such as job design, work environment, and organizational culture, may impact productivity. This indicates that we need to move beyond a narrow focus on cognitive factors and recognize the broader contextual factors that influence productivity.

Finally, the study highlights the potential of ISM analysis as a tool for understanding the relationship between causal factors of unsafe worker behavior. Using ISM analysis, the study identified the root causes of unsafe behaviour.

## Conclusions

Unsafe behavior has been identified as major contributor to the increasing spate of accidents and fatalities on construction projects. The increasing incidence of unsafe behavior and the impact thereof on construction worker productivity further accentuates the need for this A total of 15 causal factors were identified from the literature. A mix of ISM and MICMAC analysis was used to analyse data elicited from semi-structured interviews with a cohort of purposively selected and recruited experts. Using the analysis, we determined the nature of the complex relationships and dependencies among these causal factors. A mutually reinforcing and independent relationship existed between these causal factors, which indicated their reciprocal influence as well as their independence. Five levels of hierarchy were established using the ISM analysis. It was discovered that age, sleep quality, degree of interaction and workers’ skillsets were the root factors influencing the incidence of unsafe worker behavior in the study context. Several factors were identified as influencing unsafe worker behavior, including training and experience, physiological behavior, work environment, degree of interaction, worker’s skill type and sleep quality. Judging from the diagraph, sleep quality was discerned as undoubtedly a crucial factor influencing the incidence of unsafe worker behaviour. By establishing and addressing these root factors, a safe and supportive work environment can be facilitated thereby leading to improved worker productivity.

## Data Availability

The raw data supporting the conclusions of this article will be made available by the corresponding author, without undue reservation.
